# Supplementary material for: Complexome profiling of the Chlamydomonas psb28 mutant reveals TEF5 as an early PSII assembly factor
Source: Plant Cell. 2025 Mar 17;37(6):koaf055. doi: 10.1093/plcell/koaf055 (PMC12164590; doi:10.1093/plcell/koaf055)
Supplement: koaf055_Supplementary_Data [file koaf055_supplementary_data.zip › psb28_interactive.zip!/plots/intensity/qualityPlots/peptides/THI4_Cre04.g214150.t1.1.html]

### Cre04.g214150.t1.1

Trivial name: THI4  
SeqCoverage [median]: 58.00  
Mass: 36.89 kDa  
  
 

| Sequence | MissedCleavage | Mass [Da] | wtIntensity | muIntensity | Score |
| --- | --- | --- | --- | --- | --- |
| AAHMAVAALER | 0 | 1138.59 | 8881.09 | 10481.17 | 108.43 |
| AGLPPTTTPYDDYK | 0 | 1537.73 | 0.00 | 2157.67 | 101.93 |
| AGLPPTTTPYDDYKFAPIR | 1 | 2122.07 | 732.60 | 1816.87 | 32.93 |
| AGLPPTTTPYDDYKFAPIREAEVNR | 2 | 2820.41 | 0.00 | 2681.20 | 25.15 |
| ALSAAAAEGAAAEAQA | 0 | 1371.66 | 3828.90 | 12448.55 | 263.07 |
| EAEVNR | 0 | 716.35 | 0.00 | 229.21 | 84.02 |
| EVVPGMVLTGMELAEVDGSPR | 0 | 2185.08 | 5932.30 | 11109.48 | 130.05 |
| FAPIREAEVNR | 1 | 1300.69 | 2120.86 | 2901.60 | 50.28 |
| HAALLTSTLMSHVLK | 0 | 1620.90 | 739.47 | 5370.39 | 166.60 |
| HAALLTSTLMSHVLKNPNVK | 1 | 2173.20 | 0.00 | 1539.89 | 75.46 |
| KPAHEMLDALQVPYEDEGHYVVVR | 1 | 2794.37 | 0.00 | 15122.54 | 88.84 |
| LFNATAVEDLIVKPDPALGPGGRR | 2 | 2505.37 | 903.79 | 5113.32 | 34.35 |
| LGMVPGGEVPGMGALDMEAAEGSIVNNTR | 0 | 2871.36 | 2578.38 | 3631.83 | 80.36 |
| MGPTFGAMIVSGMR | 0 | 1453.69 | 5106.39 | 8916.38 | 133.05 |
| RALSAAAAEGAAAEAQA | 1 | 1527.76 | 1849.82 | 2473.38 | 130.19 |
| VAQPMSPSSAATAAADASMLAR | 0 | 2103.01 | 3160.23 | 3552.24 | 85.52 |
|  |  |  | 35833.8 | 89545.7 |  |
